# Radiation modulates expression and related activities of c-Met protein in oral tongue squamous cell carcinoma cell lines

**DOI:** 10.1007/s00432-022-04307-4

**Published:** 2022-09-02

**Authors:** Aisha A. H. Al-Jamaei, Jan G. A. M. de Visscher, Tymour Forouzanfar, Ruud H. Brakenhoff, C. René Leemans, Arwen Stikvoort, Behrouz Zandieh-Doulabi, Marco N. Helder

**Affiliations:** 1 Department of Oral and Maxillofacial Surgery and Oral Pathology, Amsterdam UMC-Location, VUMC/Academic Centre for Dentistry Amsterdam (ACTA), PO Box 7057, 1007 Amsterdam, The Netherlands; 2 Amsterdam UMC-Location VUmc, Otolaryngology-Head and Neck Surgery, Cancer Center, Amsterdam, The Netherlands; 3 Department of Haematology, Amsterdam UMC-Location VUmc, Cancer Center Amsterdam, Amsterdam, The Netherlands; 4 Academic Centre for Dentistry Amsterdam (ACTA), University of Amsterdam and Vrije Universiteit Amsterdam, Amsterdam, The Netherlands

**Keywords:** Oral tongue squamous cell carcinoma, Tongue neoplasms, c-Met expression, Tumor invasion, Radiotherapy, Radiation effect

## Abstract

**Objectives:**

c-Met, a receptor tyrosine kinase, is involved in the growth, invasion and metastasis of a variety of cancers. In a set of cell lines from several solid tumors, a five-fold increase in c-Met expression after irradiation has been reported. This study aimed to assess if c-Met is likewise abundantly expressed in oral tongue squamous cell carcinoma (OTSCC) upon exposure to irradiation, followed by a Met-induced biological response.

**Materials and methods:**

Six OTSCC cell lines were exposed to gamma radiation doses of 2, 4, and 6 Gray. The changes in c-Met protein levels were assessed by western blot and flow cytometry. c-Met gene expression, cell migration, proliferation and cell cycle assays were performed as phenotypic readouts.

**Results:**

Irradiation resulted in upregulation of c.Met in all cell lines with different time kinetics. On average the cells displayed minimal c-Met expression on their surface ranging from 5 to 30% of total protein. Abrupt downregulation of c-Met surface expression occurred one hour after radiation but recovered 48 h post-radiation. Intracellularly, the highest level of expression was found on day 5 after radiation exposure. Irradiation induced aggressive invasive potential of the cells as determined in cell migration assays, particularly in cell lines with the highest c-Met expression.

**Conclusions:**

These results provide novel insights into both intracellular and extracellular dynamics of c-Met expression profiles upon irradiation of OTSCC cells in vitro*.* It might also suggest that radiation enhances cell migration, indicative of invasiveness, through c-Met up-regulation, at least for certain types of OTSCC cells.

**Supplementary Information:**

The online version contains supplementary material available at 10.1007/s00432-022-04307-4.

## Background

Oral tongue squamous cell carcinoma (OTSCC, anterior 2/3 of tongue) accounts for approximately 41% of all oral cancers (de Camargo Cancela et al. 2010), is characterized by an aggressive biological behavior, and nearly 40% of patients have regional lymph node metastasis in the neck at initial diagnosis (Charoenrat et al. 2003). Unfortunately, despite recent advances in combined treatment modalities, OTSCC still tends to have a poorer prognosis when compared to carcinomas at other oral sites (Rusthoven et al. 2008), mainly as patients with OTSCC are at a higher risk for locoregional recurrences after treatment (Jerjes et al. 2010).

Surgery is still considered the mainstay method of treatment for OTSCC (Zhu et al. 2019). Radiotherapy, in some cases combined with concomitant cisplatin-based chemotherapy, is applied as adjuvant treatment based on the stage of the tumor, histological findings and surgical margins. Radiotherapy is also used as primary treatment, mostly combined with chemotherapy, in patients with technically or functionally irresectable tumors when organ preservation is desirable, and as a palliative measure (Huang and O'Sullivan. 2013). These aspects point to the critical role of radiotherapy for patients with OTSCC. However, the efficacy of this mode of treatment is limited by its toxicity to normal tissues (Basu et al. 2012; Yamamoto et al. 2016). In addition, radiotherapy may have unwanted side effects on the behavior of the tumor cells. Recent evidence in several types of tumors has shown that radiation treatment may actually promote the malignant behavior of the cancer cells, especially by enhancing invasion and metastasis, although the precise molecular mechanisms remain elusive (Jadhav and Mohanam. 2006; Vilalta et al. 2016). Importantly, epithelial-mesenchymal transition (EMT) has been described as an essential process concerning tumor cell invasion and metastasis (Yeung and Yang. 2017). This developmental process is primarily controlled by a network of signaling pathways that involve components such as c-Met receptor and its ligand hepatocyte growth factor (HGF) (Ohnishi et al. 2020). In this context, experimental studies have shown an association between c-Met overexpression following irradiation and enhanced invasive potential of malignant pancreatic and neuroblastoma cells (Qian et al. 2003; Schweigerer et al. 2005), while c-Met silencing or inhibition of its kinase activity abrogated this adaptive response (De Bacco et al. 2011). These data suggest a role between irradiation, increased c-Met expression and EMT resulting in an increased invasive behavior of tumor cells.

c-Met is highly expressed in head and neck cancer, presenting in up to 80%, and its overexpression has been correlated with advanced disease stage and worse prognosis (Kim et al. 2017). The overexpression of this receptor specifically in OTSCC has been shown to correlate with the enhancement of in vivo and in vitro metastasis (Lim et al. 2012). However, it is unclear whether this relates to surface expressed or cytosolic c-Met, and how expression relates to irradiation. Thus, this study was designed to reveal the dynamic changes of c-Met expression following irradiation in the sub-compartmentalization (intracellular or within the cell membrane) of the receptor for six cell lines of OTSCC. It has also examined whether c-Met expression results in a shift of the invasive behavior of the irradiated cells.

## Materials and methods

### Cell lines, culture conditions and irradiation

Six human oral tongue carcinoma cell lines (SCC-25, Cal-27, SCC-15, VU-SCC-120, UM-SCC-47 and VU-SCC-040) were used in this study. The first three cell lines were purchased from ATCC, while the others [VU-SCC-120, UM-SCC-47 (human papillomavirus (HPV)-positive), and VU-SCC-040] were provided by Prof. Brakenhoff lab (Cancer Center Amsterdam, The Netherlands). SCC-25 and SCC-15 were routinely grown in Dulbecco Modified Eagle Medium (DMEM) and Ham’s F-12, supplemented with 10% fetal bovine serum, 400-ng/mL hydrocortisone, penicillin (100 IU/mL), and streptomycin (100 μg/mL). The other four tumor cells (Cal-27, VU-SCC-120, UM-SCC-040, and UM-SCC-47) were maintained in DMEM supplemented with 10% fetal bovine serum and 1% penicillin/ streptomycin. Cells were incubated at 37˚C in a humidified incubator containing 5% CO2 and passaged at exponential growth prior to confluence. All cells were harvested using trypsin/EDTA when 80% of confluence was reached, however, in the cell surface expression experiments EDTA only, at a low concentration of 0.02% was used.

The cell lines which were provided by the laboratory of Prof. R. H. Brakenhoff (VU-SCC-120, UM-SCC-47 and VU-SCC-040), were authenticated at his lab by PCR profiling and TP53 sequencing. HPV status was confirmed by a GP5 + /6 + DNA PCR (Nagel et al. 2013). The cell lines SCC-25, Cal-27 and SCC-15 were purchased from ATCC specifically for this project and used well within 10 passages for the experiments described in the manuscript. Conform ATCC guidelines, authentication testing was, therefore, not performed in the time frame of this research project. Cells were irradiated at room temperature in a Gammacell^®^ 220 Research Irradiator (MDS Nordion, Ontario, Canada) at a dose of 2, 4 or 6 Gy (Gy).

### Western blot analysis

To determine the protein levels, cells (1 × 10^6^) were lysed on ice with RIPA lysis buffer before radiation, or 1-h, 24-h, 48-h, and 5 days after radiation with 4 Gy. Protein concentrations were measured with BCA protein Assay Kit (Pierce Chemical Co., USA), and 20 μg from each sample was separated on an SDS-PAGE gel and transferred to a PVDF membrane by electroblotting. After blocking the membrane with 5% nonfat dry milk in TBS with Tween, it was incubated with the primary antibodies: Rabbit- anti Met (1:1000; Cell Signaling, #8198), rabbit- anti phospho Met (Tyr1234/1235) (1:1000; Cell Signaling Technology, #3077), and mouse and rabbit anti- β-actin (1:1000 Abcam, ab8277 and ab6709). Subsequently, the membranes were incubated with secondary goat-anti-mouse and goat-anti-rabbit immunoglobulins (IRDye 680RD and 800 CW; Li-Cor Biosciences). Detection of bound antibodies was analyzed with an Odyssey infra-red imaging system (Li-Cor Biosciences). To evaluate the potential of c-Met to be phosphorylated in response to hepatocyte growth factor (HGF), the cells were stimulated with 50 ng/ml HGF (Thermo Fisher Scientific, Ghent, Belgium) for 10 min immediately before lysis.

### Flow cytometry

Cells were incubated at the optimal concentration in 6-wells plates. After 24 h, the cells were irradiated with a single dose of 4 Gy. The cells were rinsed with PBS and harvested at five-time intervals (pre-, 1-h, 24-h, 48-h, and 5-days post-irradiation), using cell dissociation buffer and collected into tubes containing complete media on ice. After two rinses with Cell Staining Buffer (PBS with 1% BSA) cells were ready for staining. For extracellular staining, cells were incubated with Alexa Fluor 488 conjugated rabbit-anti Met (1:100; Cell Signaling, #8494) for 30 min at 4 °C in the dark. For intracellular staining, cells were fixed in cold 2% paraformaldehyde for 15 min at room temperature and permeabilized in cell staining buffer containing 0.25% saponin (Sigma-Aldrich) for 30 min at room temperature. After two rinses with Cell Staining Buffer, cells were incubated with Alexa Fluor 488 conjugated rabbit-anti Met (1:100; Cell Signaling, #8494) for 30 min at 4 °C in the dark. Flow cytometry data were acquired using the BD FACSCelesta and analyzed with FlowJo™ Software (Tree star, Ashland, OR, USA).

### RNA isolation and real Time PCR

Total RNA was isolated using Trizol reagent (Thermo Fisher Scientific) and 750 ng of total RNA was used for First Strand cDNA using Revert Aid First Strand cDNA Synthesis Kit k1612 (Thermo Fisher Scientific), both according to manufacture instructions.

Real-time PCR on 5 × diluted cDNA was performed with a Roche LightCycler 480 II device using Cybergreen I Mastermix (Roche). The primer sequences, annealing temperature, and the sizes of amplified fragments of primers are listed in Table 1. Standard dilution method was used for the quantification of the expression of each gene. Relative gene expression of c-Met, HGF and Ki-67 were normalized to the normalization factor (NF) of YWHAZ and B2M housekeeping genes according to the following equation: NF = √(concentration YWHAZ * concentration B2M).

**Table 1:** Primer sequences used for PCR

Target gene		Oligonucleotide sequence	Annealing temperature (°C)	Product size (bp)
B2M	Forward	5' TCTGGCCTGGAGGCTATCCAG 3'	56	202
	Reverse	5' AGAAAGACCAGTCCTTGCTGAA 3'		
YWHAZ	Forward	5' GATGAAGCCATTGCTGAACTTG 3'	56	229
	Reverse	5' CTATTTGTGGGACAGCATGGA 3'		
c-Met	Forward	5' GTCCTGCAGTCAATGCCTCTC 3'	56	291
	Reverse	5' GTATTCATCGTGCTCTCACTT 3'		
HGF	Forward	5' TCAGCAAAGACTACCCTAA 3'	56	190
	Reverse	5' CTCCACTTGACATGCTATT 3'		
Ki-67	Forward	5' CCCTCAGCAAGCCTGAGAA 3'	56	202
	Reverse	5' AGAGGCGTATTAGGAGGCAAG 3'		

Primers used for the gene expression analyses showing the oligonucleotide sequences, annealing temperature and product size

*B2M* Beta-2 microglobulin, *YWHAZ* 14–3-3 protein zeta/delta, *Met* tyrosine-protein kinase Met, *HGF* hepatocyte growth factor

### Determination sensitivity to radiation

The clonogenic assay was performed based on Franken et al. (2006). Cells were trypsinized, counted and plated in 25 cm^3^ flasks. After 4 h of incubation at 37 °C, the flasks were irradiated at doses of 2, 4 and 6 Gy. Cells were incubated for 12–14 days. Subsequently, when colonies were visible, they were fixed using 100% ethanol, stained with crystal violet and counted manually. Only colonies consisting of 50 cells or more were scored. To determine cell viability, cells were seeded at the optimal density on 96-wells plates, grown for 24 h, and then the plates were irradiated at doses of 2, 4 and 6 Gy. Cells were incubated for 72 h and then cell viability was assessed using alamar blue (Invitrogen; Thermofischer) according to the manufacturer’s instruction. Fluorescence was measured at 540 nm using a Bio Tek Synergy ™ microplate reader (Bio Tek Instruments, lnc, Winooski, VT), and the results were analyzed using Graphpad Prism version 9.1.0.

### Cell migration assay

Cell migration was investigated using two different approaches: the scratch assay and transwell migration assay. With the scratch assay, duplicate 6-well plates were prepared with each of the six cell lines seeded in one well at a density of 1 × 10^5^/well and grown to confluence in a complete medium. A sterile 200 µl-pipette tip was used to make a scratch across each cell monolayer. Culture medium was discarded, and the cells were washed three times with PBS to remove the cell debris. Fresh medium was added to the cells, then one plate was exposed to 4 Gy radiation while the other one was used as a control. Multiple photographs were taken at 0 h and 24 h post-radiation under phase contrast microscopy with Zeen software. The migration speed was determined by calculating the area of the cell gap at the indicated times (0 h and 24 h), using ImageJ software (http://rsbweb.nih.gov/ij/index.html). Two images were used for each wound at each experimental point and the experiment was always carried out in duplicate. The results are expressed as a percentage of migration at 24 h with respect to zero time. With the transwell migration assay, cells were harvested at 80% confluency, and 10^4^ cells were seeded into the upper transwell chamber (Corning Costar, USA, 8 µm pore size) that were coated with 0.5 mg/ml collagen type I as previously described (Kim et al. 2016, 2010). DMEM supplemented with 10% fetal bovine serum and 1% penicillin/ streptomycin was added to the lower chamber. After 20 h of incubation, cells that had migrated to the lower chamber were fixed in 4% paraformaldehyde for 10 min, stained with 0.5% crystal violet and quantified using a wide-field microscope.

### Proliferation assay

To estimate the growth kinetics and proliferation rates of the 6 cell lines, a known number of untreated cells were seeded in a 6-well plate for 24 h to reach the exponential growth rate. The cells then were harvested using 0.05% trypsin–EDTA and counted with a haemocytometer. The doubling times (*T*_D_) were calculated in excel by using the following formula: *T*_D_ = ln(2)/*K*, where *K* = ln(*Nt*/*N*0)/*t*, where *Nt* is number of cells at time *t*, *N*0 is the initial starting number of cells at time 0, and *t* is time (hours).

### Cell cycle assay

To investigate the impact of radiation on the cell cycle, cells were seeded and treated with 4 Gy for 24 h. The next day, cells were washed twice with ice-cold PBS and fixed with 70% ethanol at 4˚C overnight. Then, the cells were incubated in 0.5 ml PBS containing 50 μg/ml RNase A for 30 min at room temperature. After that, PI was added to achieve a final concentration of 200 μg/ml for 30 min on ice in the dark. The resultant suspension was then subjected to flow cytometry analysis using the BD FACSCelesta and analyzed with FCS express V6 (De Novo Software, Ontario, Canada). The percentage of cells in the G0/G1, S and G2/M phases was calculated.

### Statistical analyses

Statistical analyses were carried out using unpaired Student’s *T* test or ANOVA where the data fit the parameters of the test. Data were expressed as mean ± SD, and *p* < 0.05(*), *p* < 0.01(**), *p* < 0.001(***), and *p* < 0.0001(****) were considered statistically significant. Pearson’s correlation coefficient r was also calculated by Excel software. *P* values for all experiments are indicated. All *P* values were generated using Prism-GraphPad software.

## Results

### C-Met is upregulated in OTSCC

**Fig. 1:**
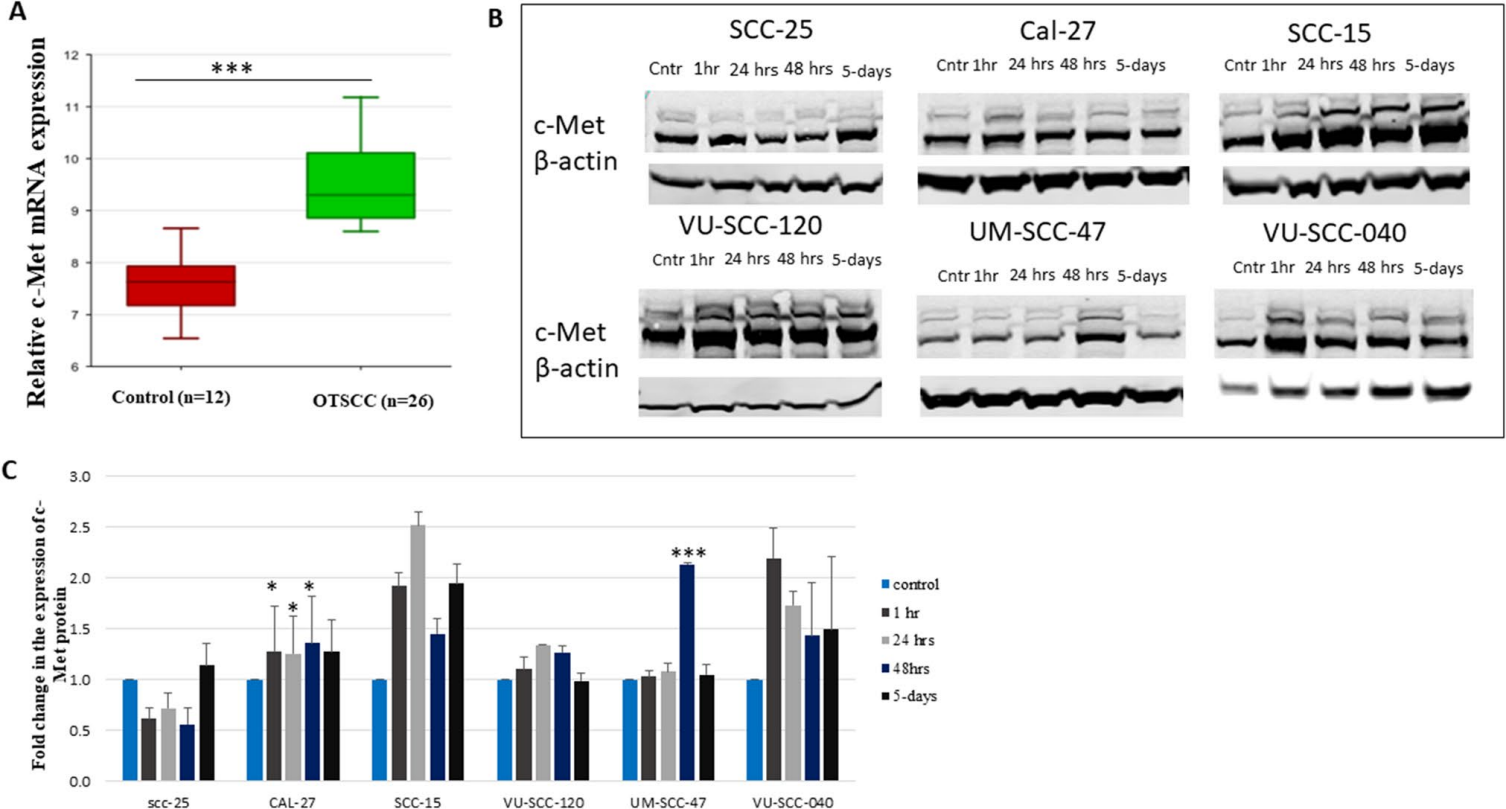
c-Met expression in OTSCC prior and after irradiation. In **A**, we analyzed a public data depository and found that the mRNA expression level of c-Met was significantly higher in the tumor compared to the normal tissue (ANOVA: *p* < 0.001). Red and green boxes represent the relative mRNA expression of c-Met in the normal and tumor samples, respectively. **B** Western blots show time-course changes in c-Met expression in a panel of 6 OTSCC cell lines. c-Met is expressed in all cells prior to irradiation (Cntr lanes). After 4 Gy, relative intensity increases clearly in SCC-15, VU-SCC-120 and VU-SCC-040. None to minor upregulation was found in SCC-25 and CaL-27. SCC-47 only showed upregulated levels at the 48-h time point. **C** Baseline expression levels of c-Met protein are distinctly lower in the UM-SCC-47 cell line. Bar graph represents the quantification of c-Met protein normalized by β-actin, representing the normalized basal expression level in each cell line (mean ± SD of triplicates). Cntr: control, 1 h:1 h, 24 h:24-h, 48 h:48-h. *p < 0.05, ***p < 0.001 compared to control. Original western blot can be found in fig.S1

As a starting point for understanding the c-Met expression profile in OTSCC, we explored its RNA expression level from publicly available data on a genomic visualization platform (https://hgserver1.amc.nl/cgi-bin/r2/main.cgi). The analysis was performed on 26 OTSCC samples and 12 normal tongue cell samples. A significant difference by one-way analysis of variance (ANOVA) was observed in favor of OTSCC (Fig. 1a). The details information about microarray analysis and samples can be found at GEO Expression Omnibus (https://www.ncbi.nlm.nih.gov/geo/query/acc.cgi?acc=GSE9844) (Ye et al. 2008).

### Radiation induced changes in c-Met protein expression

To obtain a comprehensive picture of the c-Met expression level in response to irradiation in the OTSCC, we investigated the expression of this protein at three different levels and at different time intervals: (i) the total amount of c-Met protein expressed, (ii) the intracellular c-Met expression, and (iii) the level of cell surface c-Met protein.

### Total amount of c-Met protein expressed

Western blot was performed to determine the overall c-Met synthesis after treatment with a single fraction of 4 Gy radiation at 4 time points, along with control (prior to radiation, 1-h, 24-h, 48-h, and 5 days post-radiation). All six OTSCC cell lines expressed c-Met prior and after radiation. Figure 1b and density quantifications in Fig.1c show that after exposure to the radiation and normalization using β-actin levels, four of these cells exhibited strong expression of c-Met protein (Cal-27, SCC-15, VU-SCC-120, and VU-scc-040), while the cell line of SCC-25 exhibited weak expression. It was found that VU-SCC-040 peaked to approximately 2 times the level of pre-radiated control cells at 1-h after radiation, while SCC-15 peaked at 2.1 times of untreated cells at around 24 h. With regard to the HPV + cell line (UM-SCC-47), the c-Met level remained unchanged up to 48 h, when it almost doubled, suggesting that radiation influences the expression level of the c-Met.

### Intracellular c-MET expression

**Fig. 2:**
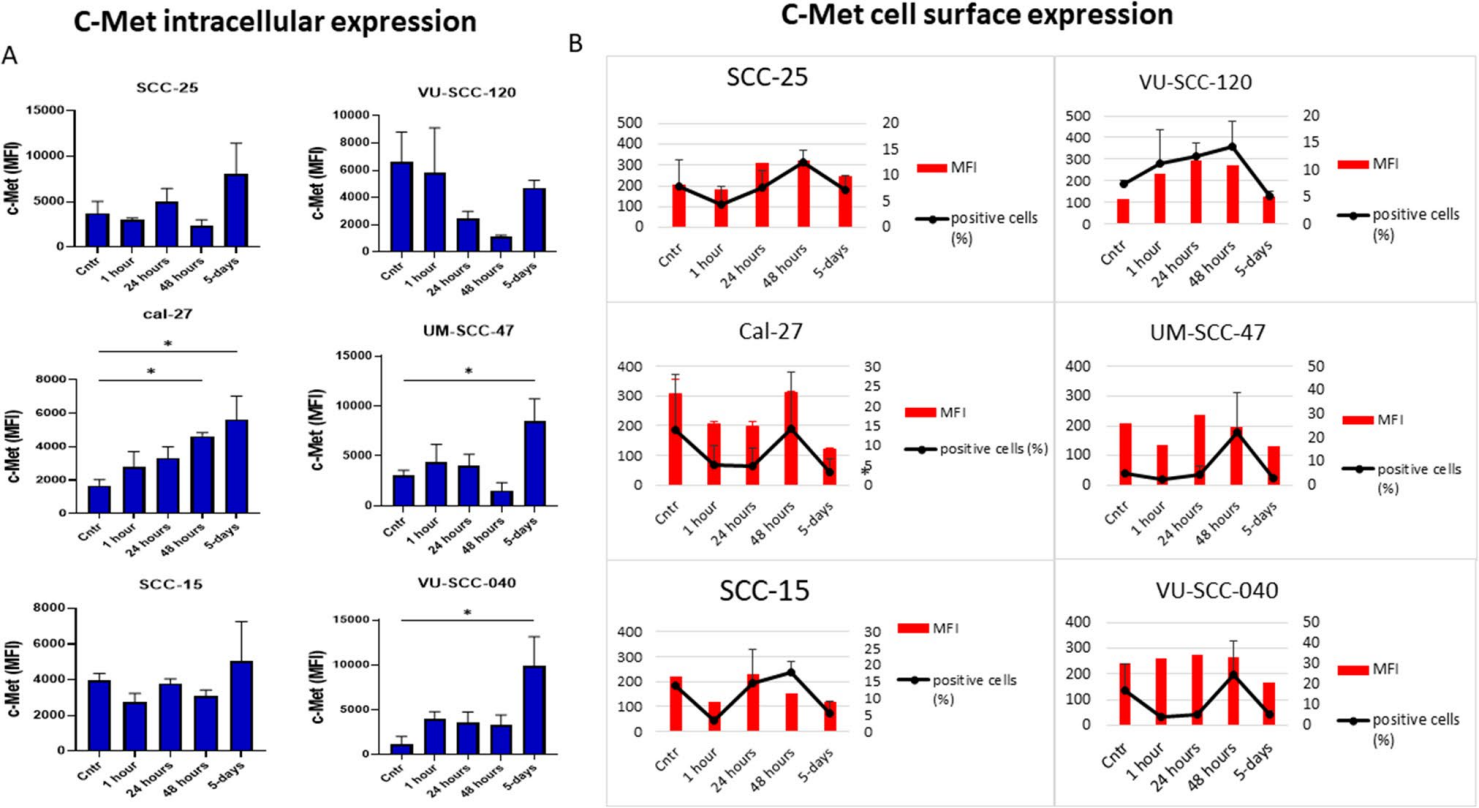
Flow cytometry data showing dynamic alterations in the intracellular and extracellular expression of c-Met protein after 4 Gy of irradiation in the OTSCC panel of cell lines, quantified as mean ± SD of duplicates. **A** Intracellular median fluorescence intensity (MFI) patterns are variable at the 1–48 h time points (increase compared to control levels in CaL-27 and VU-SCC-40, decrease in VU-SCC-120, and fluctuating around control levels in SCC-25, SCC-15 and UM-SCC-47), but consistently show an increase between the 48 h and the 5 days/120 h time point. **B** Indicated MFI and percentages of positive cells for c-Met expression on the cell surface. In all cell lines except VU-SCC-120 (which shows a gradual increase until 48 h), a decrease in the percentage of cells positive for c-MET surface expression was observed after 1 h post-radiation, with a recovery to similar (SCC-25, CaL-27, SCC-15, VU-SCC-040), or elevated levels (VU-SCC-040) compared to control after 24–48 h. From 48 h on, a consistent decrease of surface expression levels was seen to values similar or even lower than control values. Similar patterns were found (but with a lesser drop in intensities for VU-SCC-040 at 1 and 24 h compared to figure b, and no marked changes for UM-SCC-47) when assessing MFI. Cntr: control. Data are representative of no less than two independent experiments for each cell line at each time point with. *significant difference, *p* < 0.05

Flow cytometry analysis of intracellular c-Met expression showed only one population and consequently, median fluorescence intensity (MFI) was used as the qualitative measure for c-Met profiling changes. We observed that the intracellular profile of c-Met in the six cell lines showed a peak induction at around 5 days after exposure to irradiation. Meanwhile, we noticed that the 48-h time point was the common time between these cell lines where they showed downregulation of c-Met protein, with the exception of Cal-27 cells (Fig. 2a).

### Cell surface detection of c-MET

The panel of cell lines invariably showed two populations (positive and negative c-Met surface expression) on flow cytometry analysis. To gain insight into the dynamic changes of the surface expression levels, we assessed the percentage of cells that were positive for c-Met on their surface, as well as their MFI. In Fig. 2b we observed that the dynamics of c-Met expression were almost similar in five of the cell lines (SCC-25, Cal-27, SCC-15, UM-SCC-47, and VU-SCC-040). The exposure to radiation induced a striking reduction in the percentage of the positive cells at around the 1-h time point, but then gradually increased and reached the peak at 48-h’ time point. Analogous increases in MFI values at that time point were detected only in two of those cell lines (Cal-27, and SCC-25). The picture was different for the VU-SCC-120 cell line, in which a gradual increase in percentage of the positive cells and correspondingly a gradual uprising in MFI intensity was observed 1-h after irradiation and reaching a peak at 48-h (Fig. 2b). Importantly, although 48-h’ time point was a common time interval, where all cell lines showed the highest percentage of c-Met expression after radiation exposure, the percentage did not exceed 30% overall. Gating and other details are provided in supplementary data (Supplementary file 1: Fig. S2).

### Gradual increase in c-Met RNA expression level

**Fig. 3:**
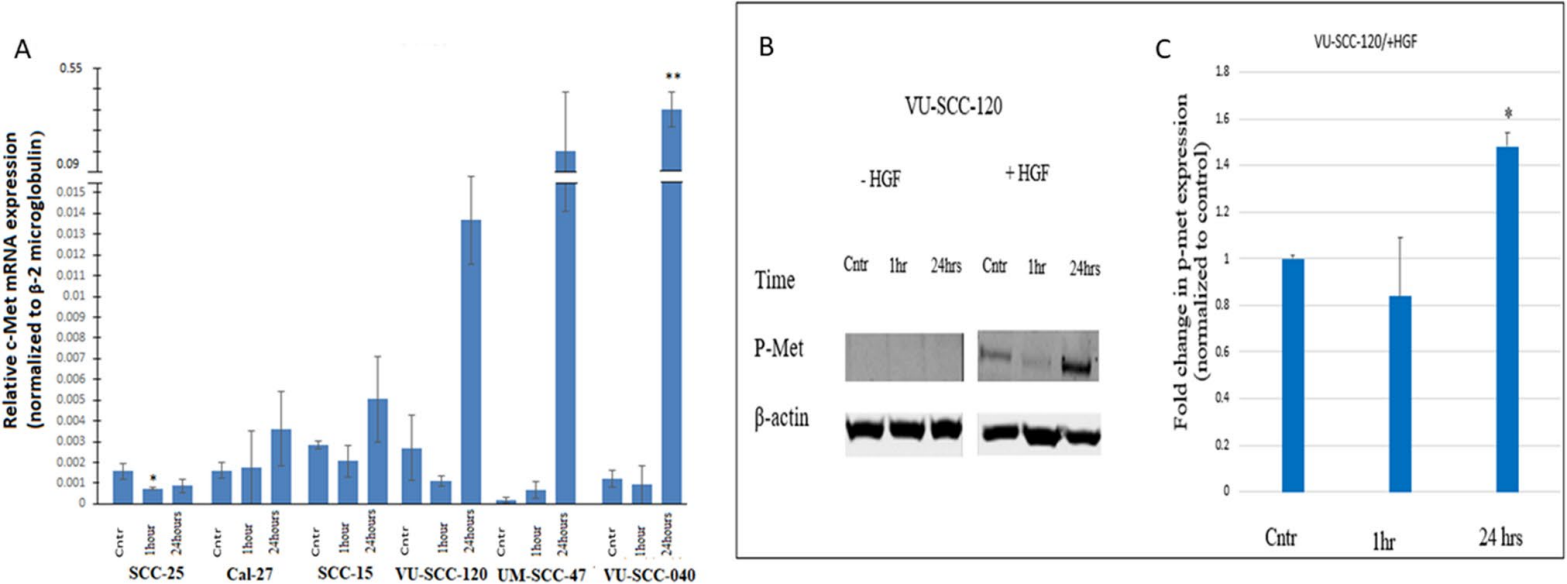
qRT-PCR result for c-Met RNA show increased expression in all cell lines except SCC-25 at 24 h after radiation exposure (**A**). The relative expression level of c-Met was normalized to Beta-2 microglobulin, and YWHAZ: 14-3-3 protein zeta/delta as housekeeping genes. HGF-triggered c-Met tyrosine phosphorylation for VU-SCC-120 cell lines (**B**). The p-Met expression was detected by western blot analysis. The amount of p-Met was quantified and normalized against β-actin and the ratio of normalization was shown in **C**. Bar graph represents mean ± SD of three independent experiments. + HGF: with adding HGF; − HGF: Without HGF. Cntr: control, 1 h:1 h, 24 h: 24 h. *p < 0.05, **p < 0.01 vs control. Original western blot can be found in Fig. S2b

The noticeable quantitative alterations in the intracellular and extracellular protein expression led us wondering whether those changes were reflections of modulated localization of this receptor or a result of RNA synthesis. Hence, to verify the observed difference in the protein level at different time intervals, we next evaluated the gene expression of c-Met, and its ligand (HGF). Three-time intervals in accordance with the time chosen for protein analysis were selected to investigate the correlation of gene and protein expression. The results (Fig. 3b vs. Fig. 1b) revealed that gene expression was not in accordance with the protein levels at the indicated time points. Nonetheless, the highest gene expression for the c-Met was noticed at 24-h which may indicate gradual upregulation of the gene with time after irradiation. However, the opposite results were noticed for the SCC-25, in which the level of the c-Met RNA downregulated significantly from untreated at 1-h and markedly diminished at 24-h (Fig. 3a).

### HGF is not secreted by OTSCC cells

Regarding HGF, there is considerable debate whether the cancer cells secrete this growth factor or if this is done by stromal cells such as fibroblasts. In our analysis, HGF mRNA was not detected in any of the OTSCC cell lines, providing further evidence that this growth factor is likely to be secreted by cancer-associated fibroblasts (CAF). Nonetheless, it is also possible that the HGF amount is too small to be detectable by our technique (data not shown).

### C-Met phosphorylation is functional, but only occurs upon HGF stimulation

c-Met phosphorylation has been reported to be induced upon exposure to irradiation in the absence of HGF. We, therefore, performed a western blot to detect p-Met (Tyr1234/1235) in the absence and presence of its selective ligand (HGF). We found that lack of p-Met expression was a constant finding in all cells at all-time points in case of the absence of the ligand (data not shown). However, with the addition of HGF to the VU-SCC-120 cells—this cell line was chosen because it displayed the highest c-Met expression at baseline and showed high expression after irradiation (Fig. 1b, c)—more phosphorylation at 24-h post-irradiation was clearly observed in comparison to the early time-point (1 h) post-irradiation (Fig. 3b, c). This suggests that the presence of the ligand is necessary for functional c-Met in OTSCC. Additionally, this seems consistent with findings by De Bacco et al. which suggested that in irradiated cells c-Met expression became more sensitive to HGF stimulation (De Bacco et al. 2011).

### Various c-Met expression levels cannot explain innate sensitivity of the cells to the radiation

**Fig. 4:**
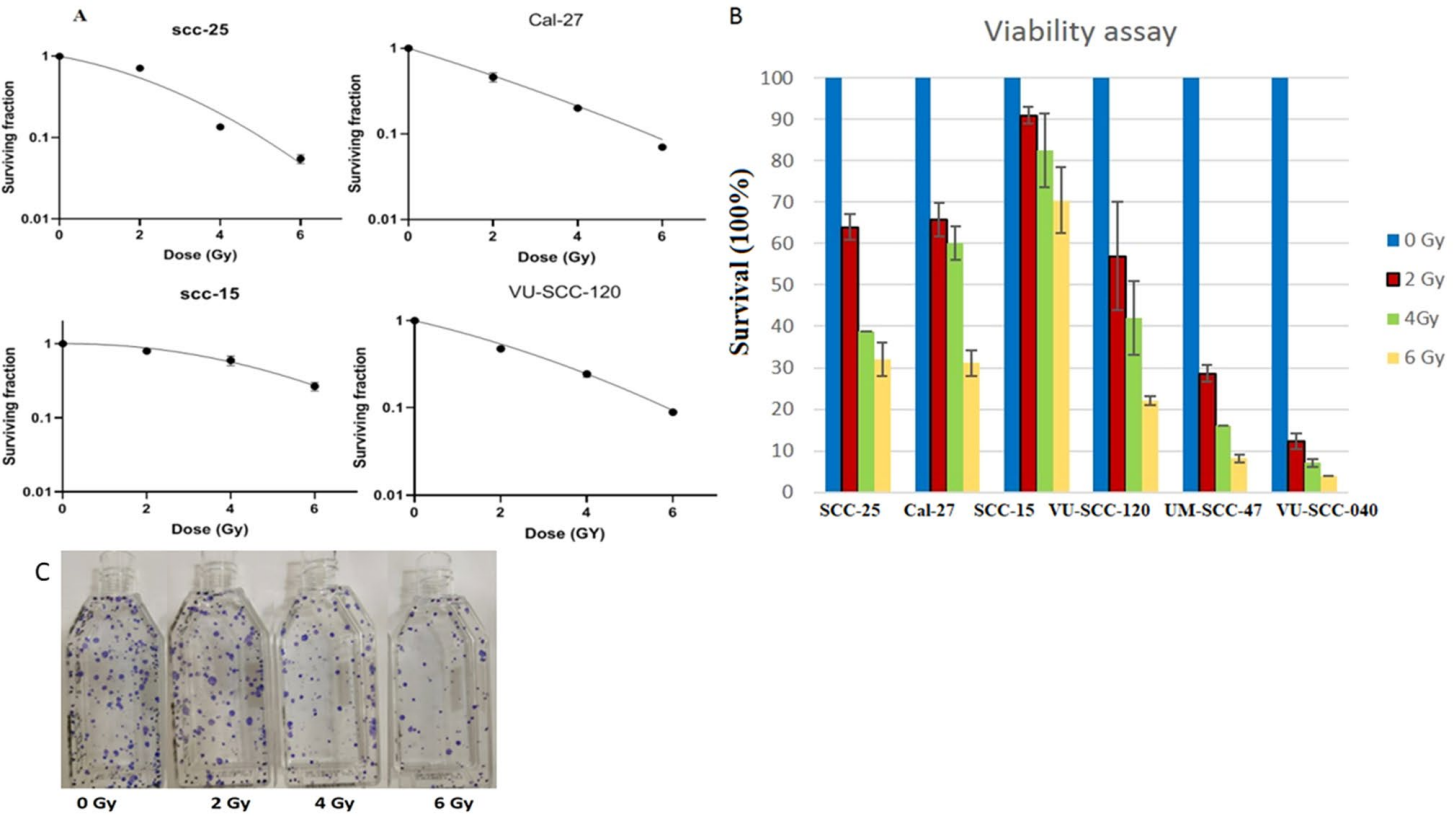
Determination of cell viability of a panel of OTSCC cell lines in response to irradiation as assessed by clonogenic assay (**A**) and Alamar blue (**B**). representative picture of the clonogenic assay for Cal-27 cell line; colonies stained with crystal violet and counted manually after 12–14 days of radiation exposure (**C**). We found that UM-SCC-47 and VU-SCC-040 could not grow in colonies, so these cell lines are missing in Fig. 5 A. Values in the bar and line graphs represent mean ± SD of 3 experiments

It is currently accepted that c-Met contributes to the acquisition of resistance to radiotherapy in some tumors. To link the assessment of the protein in the previous section to the character of the cells either being radio-sensitive or -resistant, clonogenic and viability assays were performed on all cell lines of our panel (Fig. 4). The clonogenic assay is considered as the gold standard for radiosensitivity evaluation, but because two cell lines (UM-SCC-47 and VU-SCC-040) were not able to grow in colonies, we further explored the survival of irradiated cells by viability assay. In line with several studies, the results were comparable for both assays. Both the alamar blue assay and clonogenic assay showed that SCC-15 cells were the most radioresistant whereas VU-SCC-040 cells were the most radiosensitive (Fig. 4a, b). To further investigate the correlation between c-Met expression level and radiosensitivity, a linear regression analysis was generated for the cell lines that were clonogenic. Linear regression analysis of survival fraction 2 (SF2) values versus c-Met values revealed a moderately positive, but non-significant correlation between c-Met level and radiosensitivity (*r* = 0.52, *P* = 0.47), indicating and supporting that other factors are more likely to influence the sensitivity of the OTSCC cells to radiation.

### Correlation of c-Met expression with invasion

**Fig. 5:**
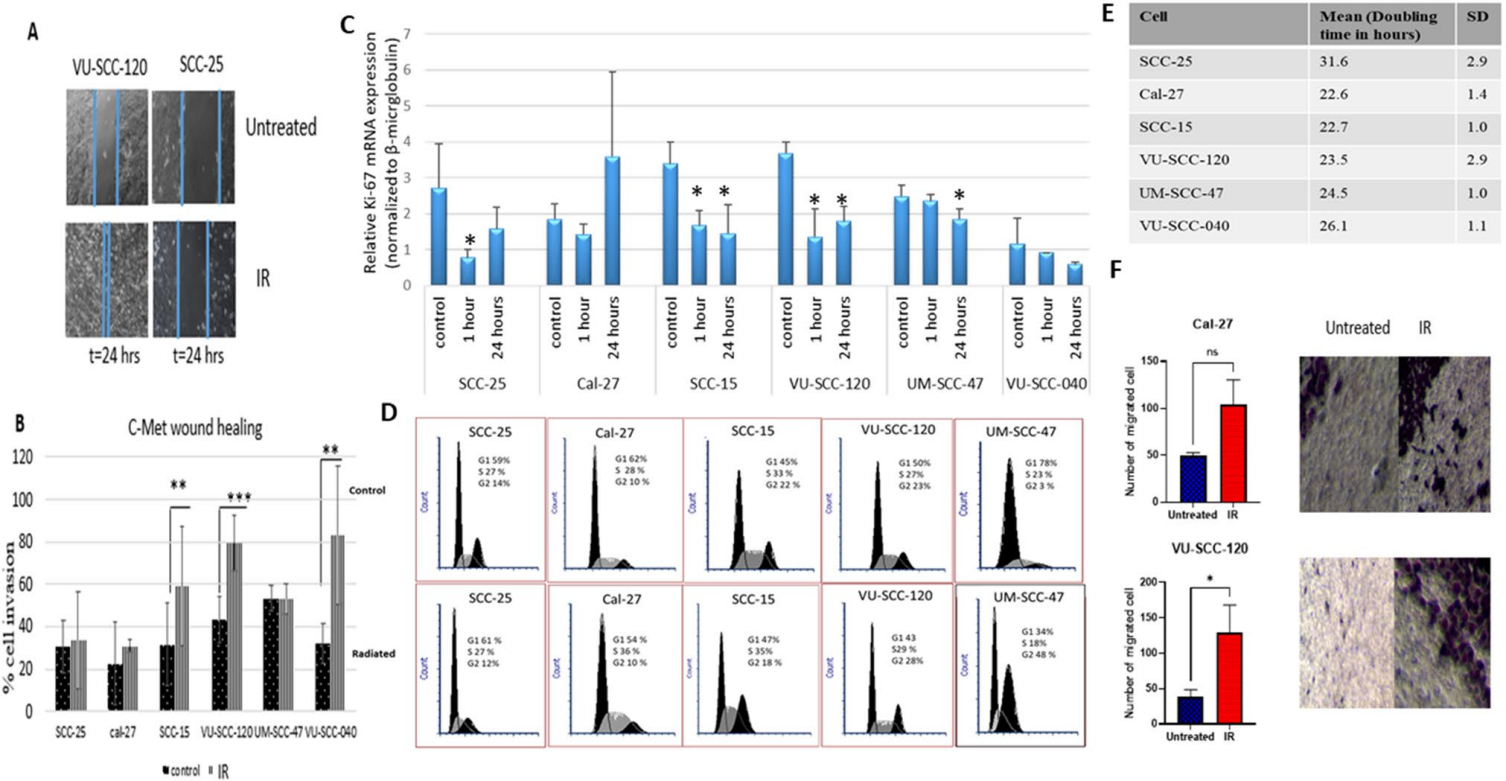
Lack of a correlation between proliferation rates and invasiveness as assessed by a scratch/wound healing assay. **A** The wound healing assay reveals that the cells showing high overall c-Met expression are the cells also showing a higher percentage of invasion ability (VU-SCC-120, VU-SCC-040 AND SCC-15). Depicted are representative pictures of the cell line with high invasive potential (VU-SCC-120) and low invasive potential (SCC-25). Bar graphs represent the mean ± SD (*n* = 3) (**B**). (**C**) Relative Ki-67 mRNA expression was assessed by qRT-PCR and showed a decrease of 24 h after radiation in all cell lines except Cal-27. Cell cycle analysis was performed by flow cytometry and showed the highest proportion in s-phase and G2/M-phase for Cal-27 and UM-SCC-47 after irradiation, respectively **D**. In **E** we calculated the doubling time in hours of the 6 studied cell lines. Transwell migration assay was performed on two cell lines as shown in representative pictures and bar graphs (**F**). Data are mean ± S.D of three independent experiments. * *p* < 0.05, ** *p* < 0.01, *** *p* < 0.001 vs control. *NS* non-significant, *IR* irradiation

c-Met has a regulatory function in cell motility and invasion, and to assess these functions, we performed an in vitro wound healing assay in all cell lines. In accordance with c-Met protein expression, the cell lines with strong overexpression (VU-SCC-120 and VU-SCC-040) covered the wounded area more readily than the cell lines with weak or stable expression of c-Met (Fig. 5a, b). Pearson correlation analysis was subsequently conducted to explore the potential correlation between the closure of the gap and c-Met protein expression. A positive, but the non-significant correlation was detected (*r* = 0.4, *P* = 0.116).

Of note, cell proliferation can compete with cell migration to close the gap of the scratch. To improve our understanding of whether the closure of the gap after irradiation was related to increased proliferation rates, or solely related to cell motility and migration, in which both functions are enhanced with a higher level of c-met expression, we first calculated the doubling times of the 6 cell lines. We found that the SCC-25 cell revealed the slowest growth rate compared to all other cell lines (*p* < 0.0001, Mann–Whitney *U* test) (Fig. 5e). No correlation between cell growth and gap closure was detected (*r* = − 0.138, *P* = 0.58). To further study the role of cell proliferation in gap closure, we explored Ki-67 mRNA expression as a prominent markers per se of cell proliferation along with cell cycle profiling. As shown in the figure (Fig. 5c), compared to the control, only Cal-27 cell line revealed an increase in Ki-67 mRNA 24 h after irradiation. Likewise, in cell cycling, the highest proportion of the S- phase and G2/M phase in the radiated cells compared to the contro were observed in Cal-27 (36%) and UM-SCC-47 (48%), respectively (Fig. 5d), wherein both showed a slow rate of gap closure. Together, these results exclude any possible growth effect on the gap closure.

Next, we performed transwell migration assay to assess the capacity of two chosen cell lines (Cal-27 and VU-SCC-120) to migrate and close the wound. Those two cell lines were chosen because they showed considerable difference in their rates of gap closure (Fig. 5b). In accordance with scratch assay observations, no significant difference in migration behavior of Cal-27 before and after irradiation was detected, while the migration ability of VU-SCC-120 (cell line with strong expression of c-Met after irradiation) enhanced significantly upon irradiation (Fig. 5f). These observations suggest that c-Met plays a key role in migration and subsequent invasion of OTSCC cells.

## Discussion

c-Met is an important transmembrane receptor for regulating cancer cell migration and invasion (Arnold et al. 2017) and is closely correlated with poorer prognosis in various cancer types (Liu et al. 2012; Park et al. 2012), including OTSCC (Lim et al. 2012; Lo Muzio et al. 2004). Intriguingly, De Bacco et al. showed that ionizing radiation caused an increase in c-Met expression and increased migration activity in a set of cancer cell lines. Such an effect was ligand-independent and resulted in increased invasiveness of the irradiated cells (De Bacco et al. 2011). To the best of our knowledge, this study is the first to describe the dynamic changes in the intracellular and extracellular c-Met expression profiles and their adaptive response to irradiation in OTSCC cell lines.

Our study shows that c-Met is abundant at the total protein level, however, the fraction located on the cellular surface was rather low, not inducible by irradiation and strongly declined shortly after radiation treatment, and again at 5-day post-irradiation. The study also showed that the HPV positive cell line UM-SCC-47 exhibited a distinct expression level of c-Met and successive biological response. Furthermore, the study suggests a possible involvement of c-Met expression in irradiation-induced aggressive invasive potential in OTSCC. However, it needs to be kept in mind that this is based on a small series of cell lines and needs conformation in further investigations.

Even though western blot analysis showed abundant total c-Met protein in all cell lines, the percentage of cells expressing c-Met on their surface did not exceed 30%. We observed a strong reduction in this percentage one hour after radiation and again at 5-days post-irradiation. The phenomenon of acute downregulation of c-Met surface expression leads us to hypothesize that this might be the result of a progressive internalization of the c-Met receptor into the intracellular compartment. Abrupt removal of the receptor from the cell surface (internalization) is an essential mechanism used by the cells to prevent sustained stimulation. Internalization of receptor tyrosine kinases such as c-Met may be accelerated by ligand binding on the cell surface (Goh and Sorkin. 2013). A possible explanation for c-Met immediate internalization after irradiation might be the direct effect of the radiation itself as suggested by McRobb et al. These authors have reported induction of CD166 trans-localization from the intercellular junction into the apical surface by ionizing radiation, which could be the case for c-Met as well (McRobb et al. 2019). Independent of the ligand, internalization has also been demonstrated to be mediated by other mechanisms such as acetylation of the receptor which warrants further investigation (Goh and Sorkin. 2013).

Regarding the behavior of HPV + cell line (UM-SCC-47), these cells exhibited the lowest expression level of c-Met as native cells (Fig. 1c). After exposure to radiation, c-Met was not inducible (Fig. 1b), and also no enhancement in the malignant behavior was seen (Fig. 6a). In fact, these findings do not support an earlier study that HPV E6 significantly induces c-Met overexpression through downregulation of wild-type P53 in head and neck cancer (Qian et al. 2016). However, our data are comparable to a study by Kwon et al. that reported a significant negative association between P16 positivity, which is an indicator of HPV-related head and neck cancer, and c-Met overexpression (Kwon et al. 2014). Additionally, in a study assessing 223 locally advanced head and neck carcinoma, high c-Met protein expression was found in 73% of HPV-negative tumors, but only in 27% of HPV-positive tumors (Baschnagel et al. 2014). Admittedly, our observation is based on one cell line which is not enough to confirm the result.

It is well known that irradiation-induced c-Met overexpression involves a transcriptional mechanism. A recent study by Jahn et al. found in an in vivo model a significant correlation between upregulation of c-Met RNA and acquisition of EMT phenotypes (Jahn et al. 2012). EMT is a pivotal process for the cancer cells to acquire invasive and metastatic potential and the most important hallmarks of this event are downregulation of E-cadherin (epithelial marker) and upregulation of vimentin (mesenchymal marker) (Huang et al. 2012). In a recent study, Jiao et al. overexpressed c-Met in two lung cancer cell lines (A549 and PC-9). The investigators subsequently found enhanced EMT characteristics in these cells, i.e. a spindle shape, reduction in E-cadherin expression, and increased vimentin expression (Jiao et al. 2016). Collectively, it seems that a marked c-Met upregulation, both at the protein and RNA level facilitates invasion through inducing EMT. Consistent with these studies, our results showed that the cell lines SCC-15, VU-SCC-120 and VU-SCC-040 which exhibited higher levels of c-Met protein and RNA expression also displayed higher invasive potential. Here, it is interesting to note that the above-mentioned studies showed induction EMT and subsequently invasion behavior by canonical c-Met signaling in the presence of HGF. On contrary, as our results showed very low expression of cell surface c-Met, the correlation we observed between c-Met expression and invasion potential is likely linked to non-canonical pathways. Clearly, additional research is needed to clarify such a correlation and which non-canonical signaling is involved, if any. Moreover, our finding of variable levels of irradiation-induced invasiveness in our OTSCC panel of cell lines may partly reflect the clinical situation, since metastatic behavior after radiotherapy is reported to be occurring in only 10–25% of head and neck cancers after radiotherapy (Schaaij-Visser et al. 2010). Overall, this finding warrants further clarifications, especially for signal transduction mediators, as these may contribute to developing valuable targeted therapy to be used concomitantly during radiotherapy.

There are several limitations of our study. First, all experiments were performed in the absence of HGF, which is the only known c-Met ligand and may influence the dynamics and relative distribution of c-Met over the intracellular and extracellular compartments. In addition, it has been reported that the cells that exhibited overexpression of c-Met after irradiation became more sensitive to HGF, which subsequently enforced their malignant behaviors. With this in mind, future experiments will be performed with HGF added to the culture media. Second, we did not investigate which signaling pathways are induced upon c-Met overexpression in OTSCC and are involved in promoting cellular invasion. Unraveling these signaling pathways may provide opportunities to identify novel targets to enhance the effectiveness of radiotherapy while prohibiting the risk of inducing enhanced invasiveness. Furthermore, potent c-Met inhibitors like crizotinib or cabozantinib were not used in this study, which might have provided insights into their feasibility to preclude metastatic behavior in particular in tumor cells showing high c-MET expression and concomitant high invasiveness. Hence, the possible involvement of c-Met expression levels in irradiation-induced cell invasion should be considered preliminary.

## Conclusions

Our analysis demonstrates dynamic changes after irradiation in the intracellular and extracellular c-Met profiles of the OTSCC cells in vitro. The study suggests that c-Met is inducible upon irradiation in most of the HPV-negative cell lines, but not in HPV-positive cell lines. Irradiation also induced invasive potential in some cell lines and seems to associate with upregulation of c-Met at both protein and RNA expression that warrants further preclinical evaluation since it may have substantial clinical implications.

## Supplementary Information

Below is the link to the electronic supplementary material.

Supplementary file1 (DOCX 4230 kb)

Supplementary file2 (DOCX 652 kb)

## Data Availability

The data that support the findings of the current study are available from the corresponding author upon reasonable request.

## Abbreviations

OTSCC: Oral tongue squamous cell carcinoma
c-Met: Mesenchymal-epithelial transition factor
EMT: Epithelial-mesenchymal transition
HGF: Hepatocyte growth factor
HPV: Human papilloma virus
p-Met: Phosphorylated c-Met
