# Supplementary figures and images for: Radiation modulates expression and related activities of c-Met protein in oral tongue squamous cell carcinoma cell lines
Source: J Cancer Res Clin Oncol. 2022 Sep 2;149(8):4173–84. doi: 10.1007/s00432-022-04307-4 (PMC10349745; doi:10.1007/s00432-022-04307-4)

Figure S2: Naive western blot membrane

a)

c-Met


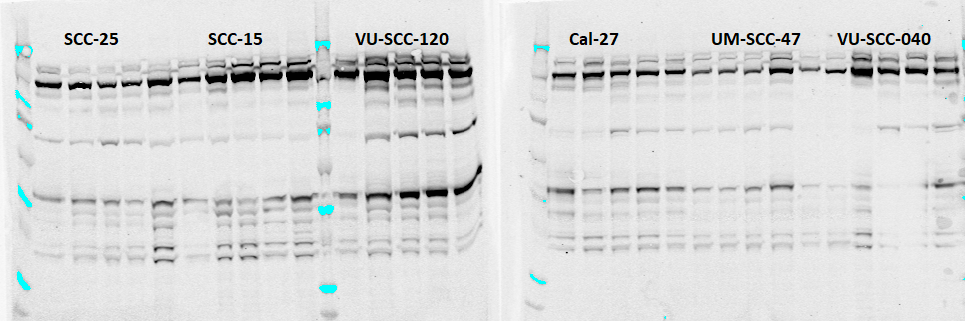




β-actin

b)

VU-SCC-120/p-Met


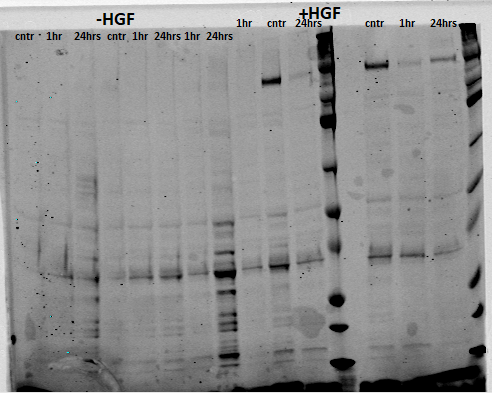

Supplement: Supplementary file 1 — Supplementary file1 (DOCX 4230 kb) [file 432_2022_4307_MOESM1_ESM.docx]
