# Supplementary material for: Radiation modulates expression and related activities of c-Met protein in oral tongue squamous cell carcinoma cell lines
Source: J Cancer Res Clin Oncol. 2022 Sep 2;149(8):4173–84. doi: 10.1007/s00432-022-04307-4 (PMC10349745; doi:10.1007/s00432-022-04307-4)
Supplement: Supplementary file 2 — Supplementary file2 (DOCX 652 kb) [file 432_2022_4307_MOESM2_ESM.docx]

**Fig.S1**: Gating strategy for flow cytometry analysis for the six OTSCC cell lines at 5 time points, A-D. A Doublet cells were excluded based on forward

scatter height (FSC.H) vs forward scatter area (FSC.A). B, cells were selected based on(FSC.A) vs side scatter area (SSC.A). C, positive cells were

selected based on (SSC.A) vs FITC. D, showed data in gray and color histogram. Percentages (%) and median fluorescence intensity (MFI) were measured.

This strategy was followed for both extracellular and intracellular analysis. Analysis for all six cell lines are shown.

A minimum of 10,000 cells was used in the analysis. 0h: control, 1h: one hour, 24h: 24 hours, 48h: 48 hours, 120h: 120 hours or 5-days.

A B C D

#
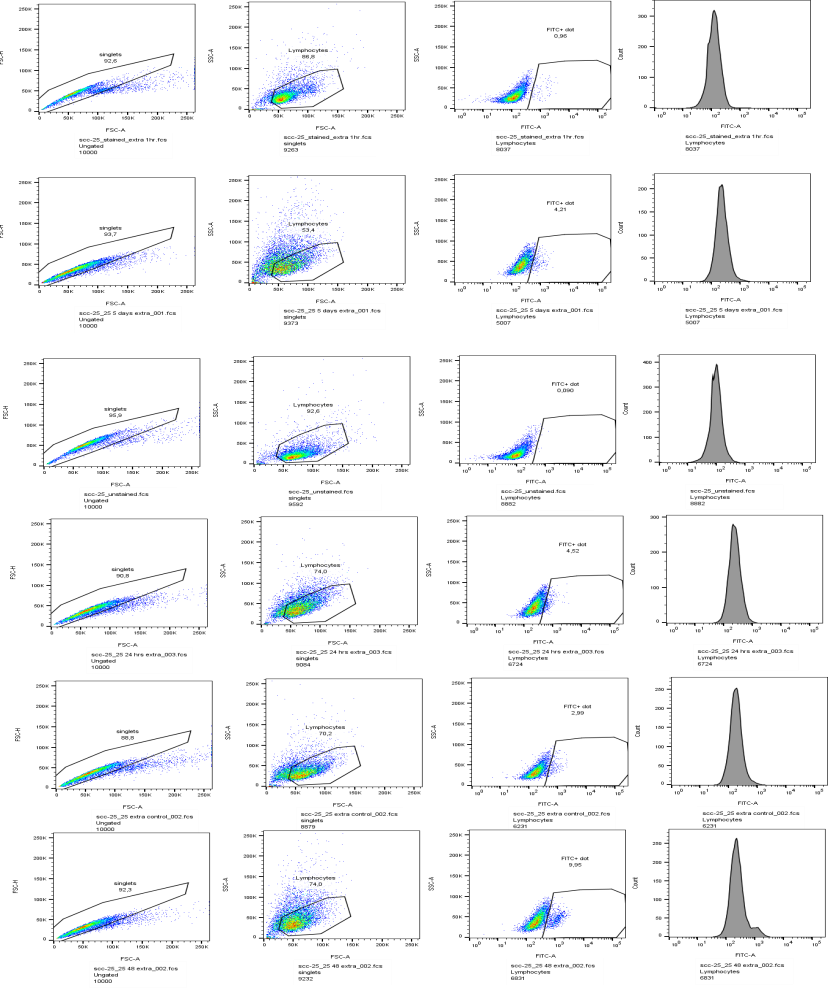
SCC-25 extracellular


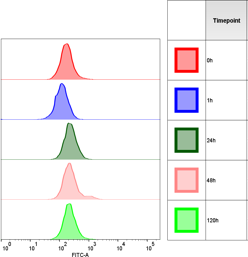


D


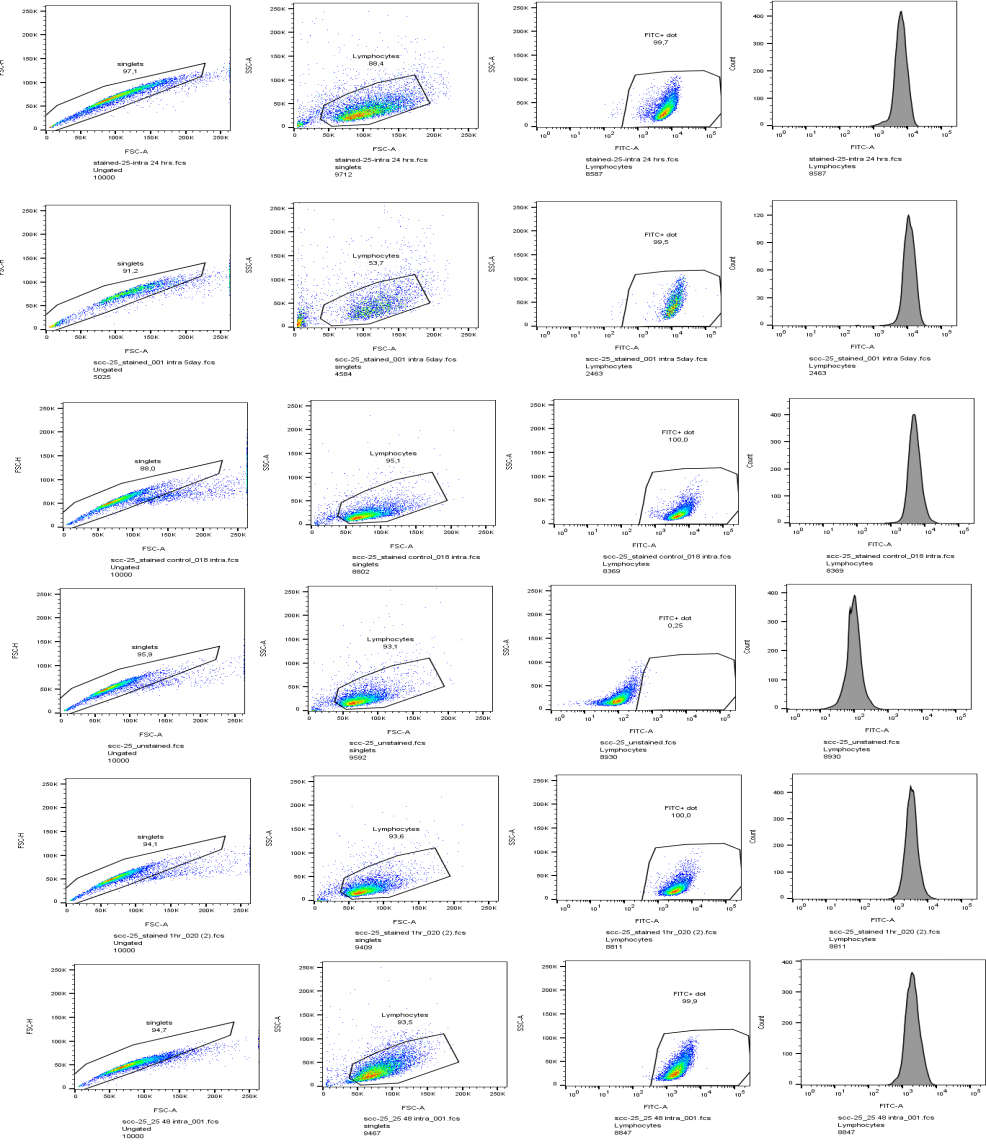
A B C D

# SCC-25 intracellular


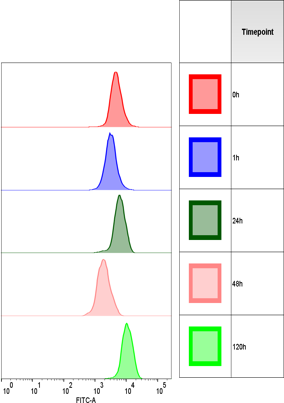


D


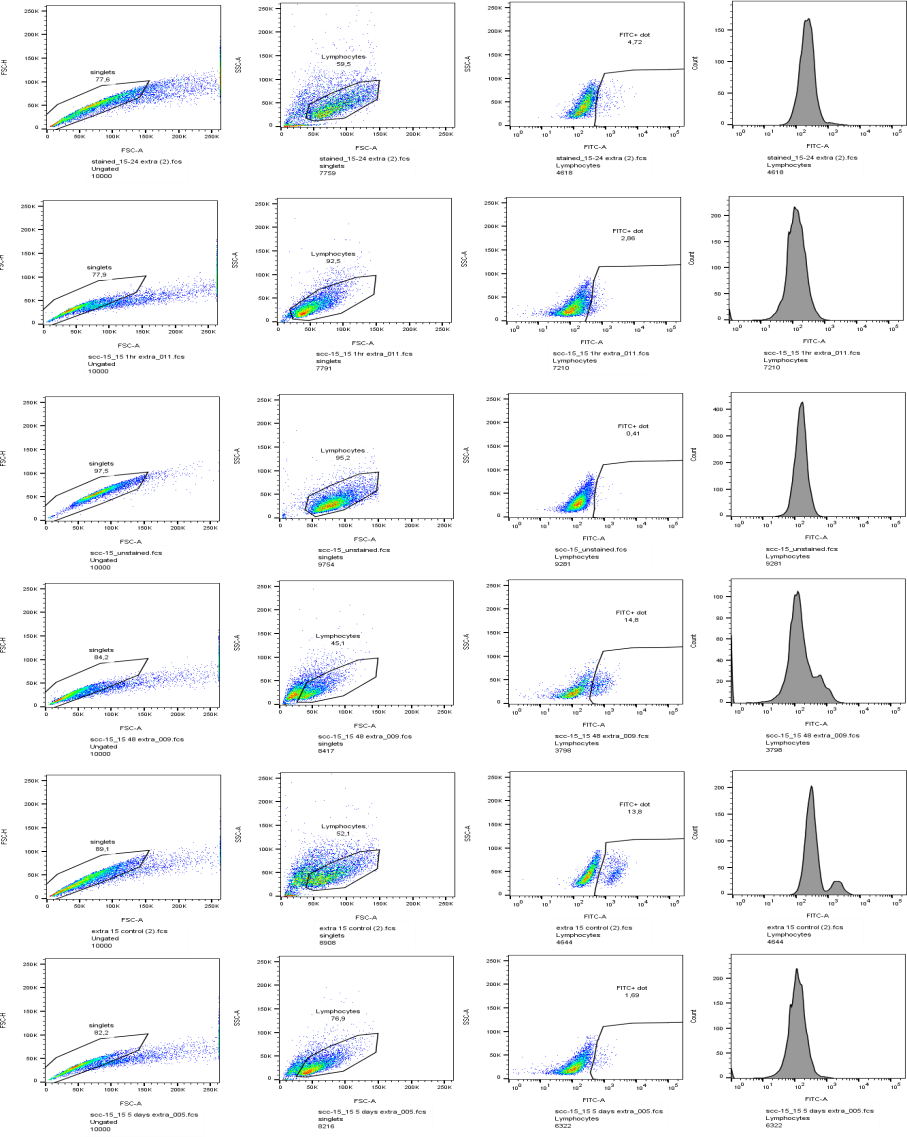
A B C D

# SCC-15 extracellular


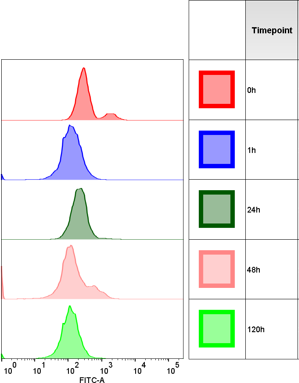


D


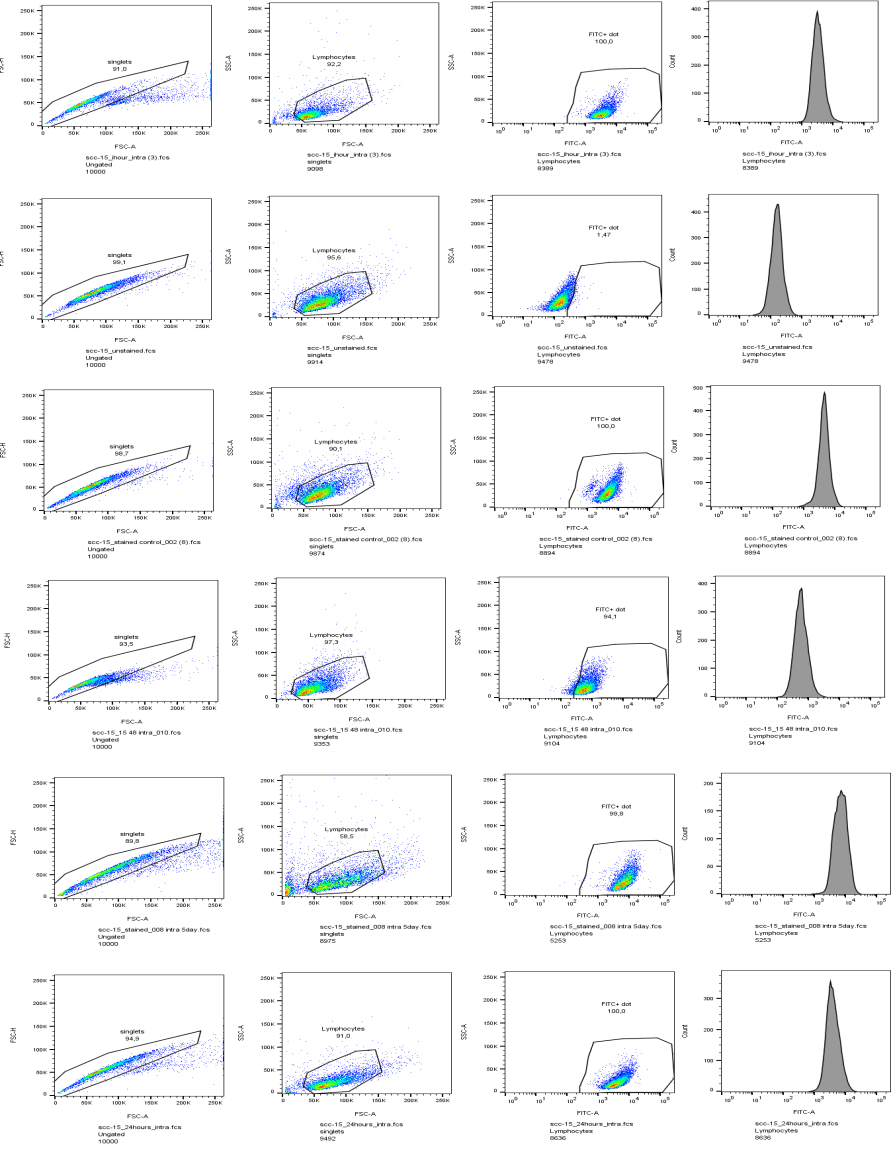
A B C D

# SCC-15 intracellular


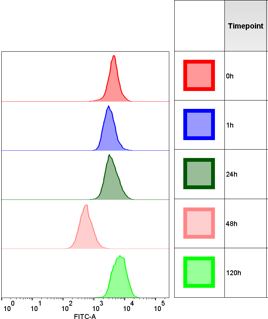


D


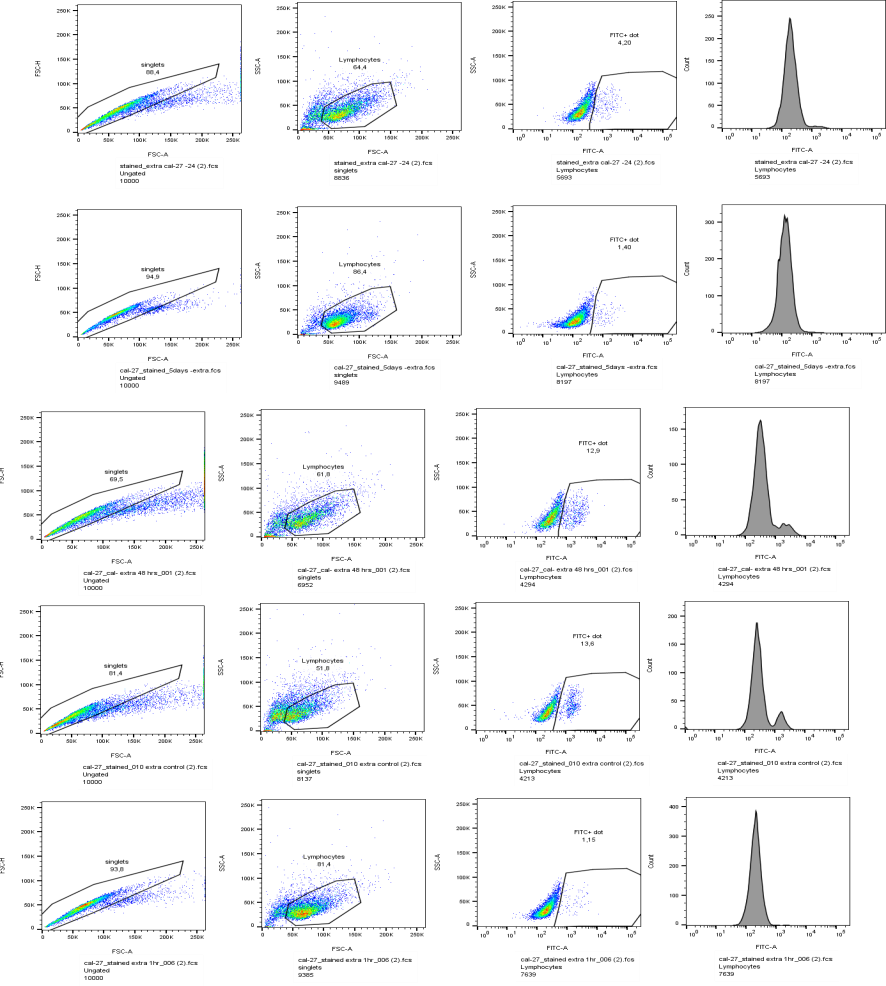
A B C D

# Cal-27 extracellular


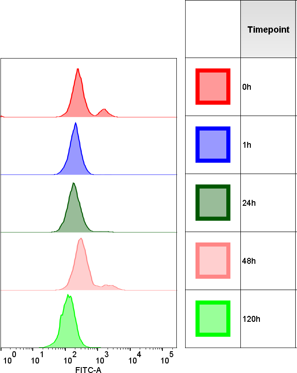


D


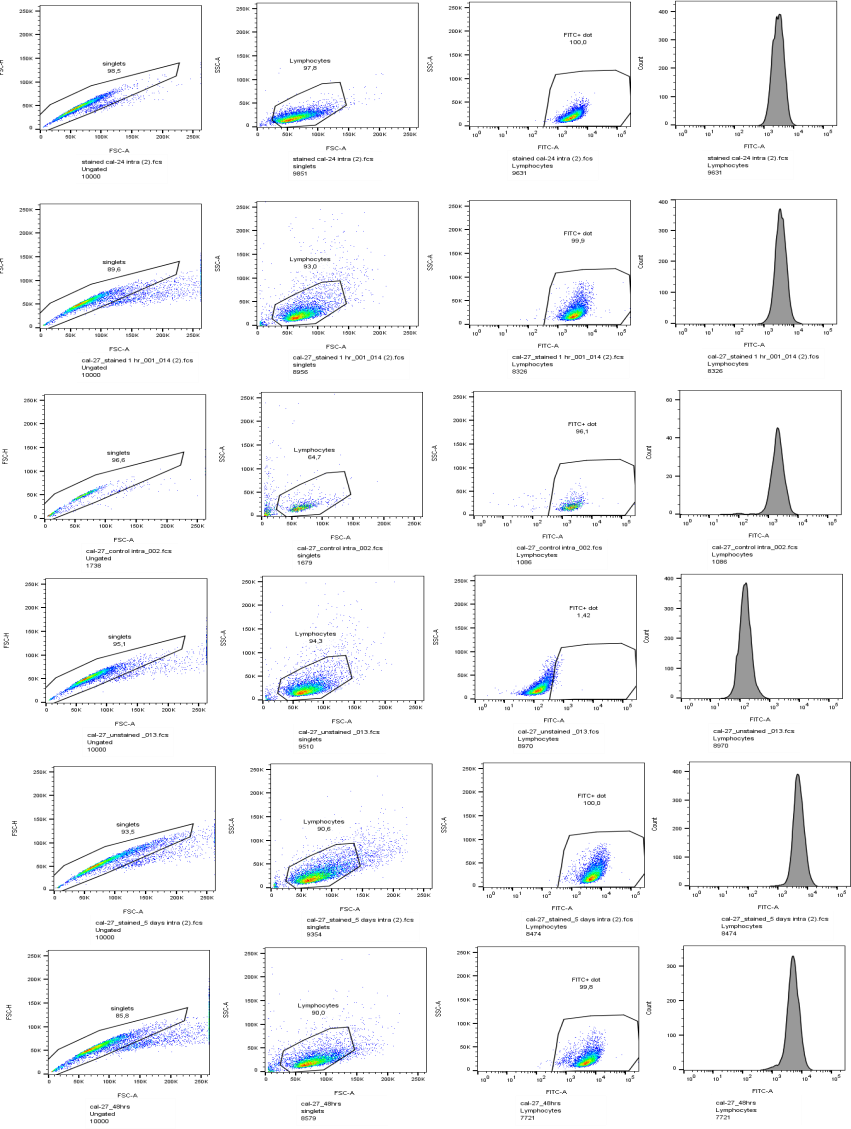
A B C D

# Cal-27 intracellular


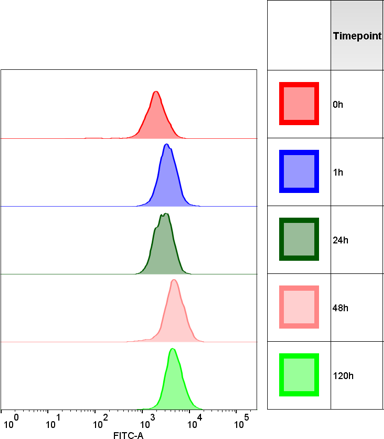


D

A B C D

# VU-SCC-120 extracellular


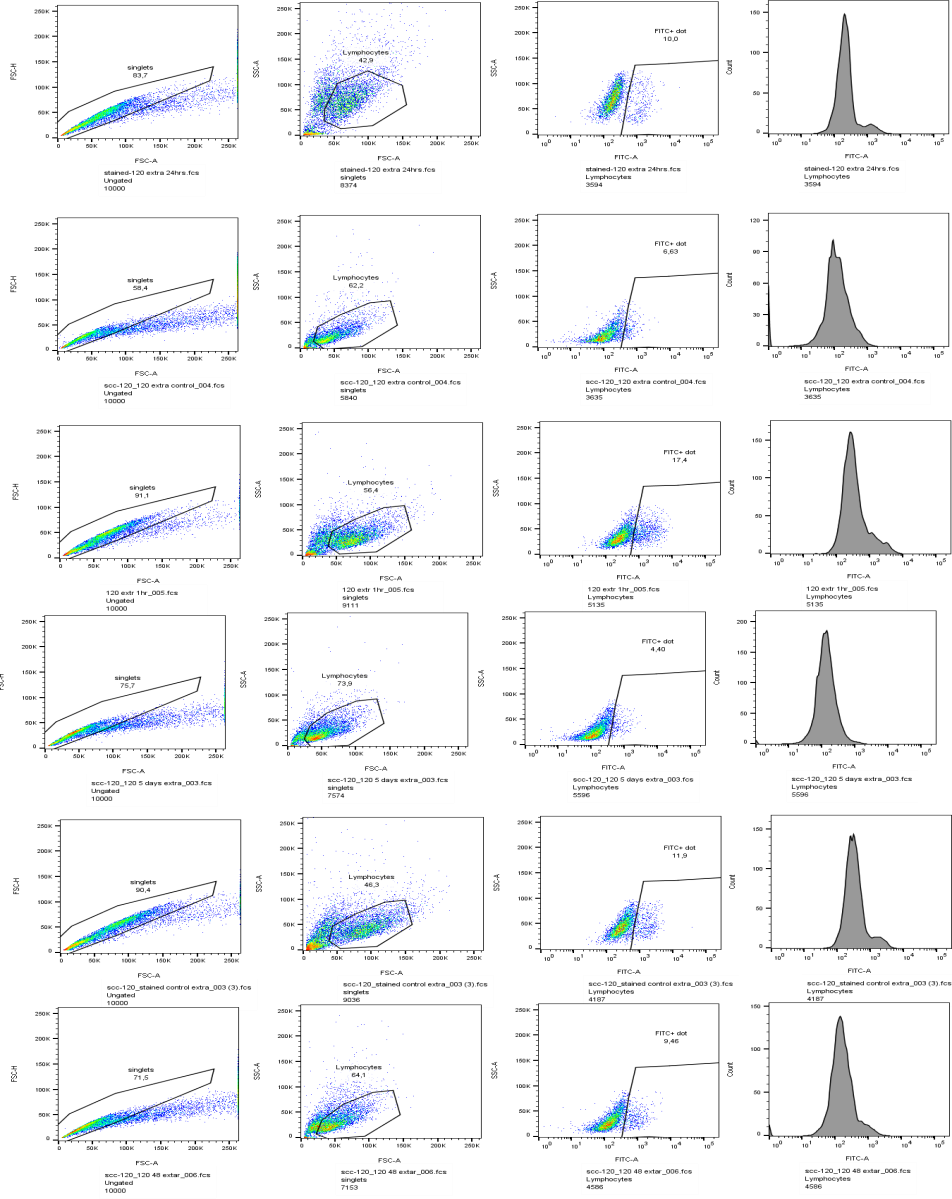


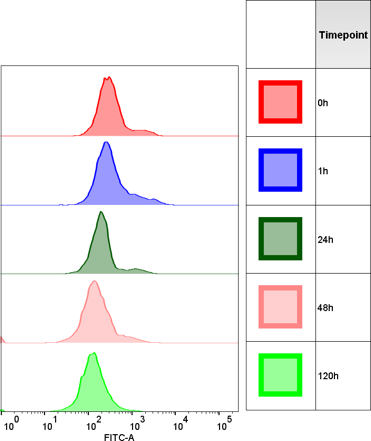


D


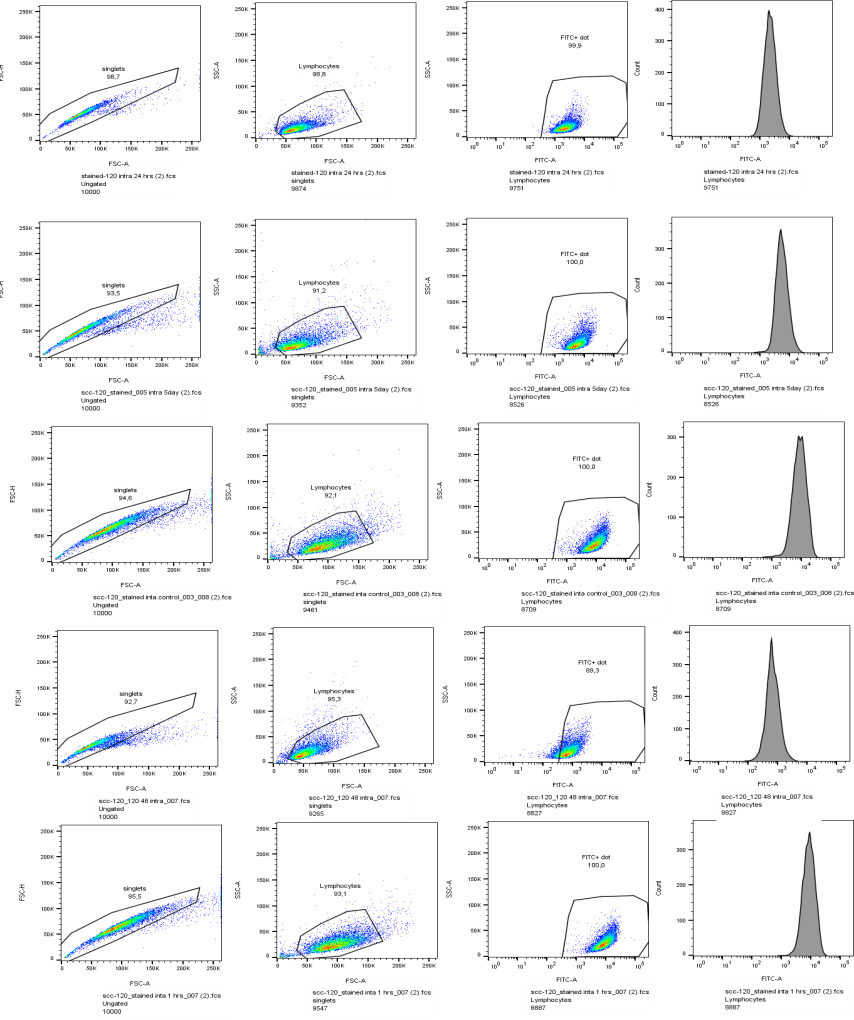
A B C D

# VU-SCC-120 intracellular


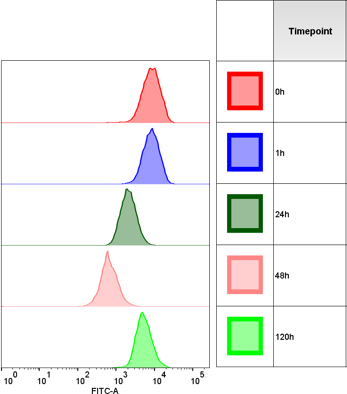


D


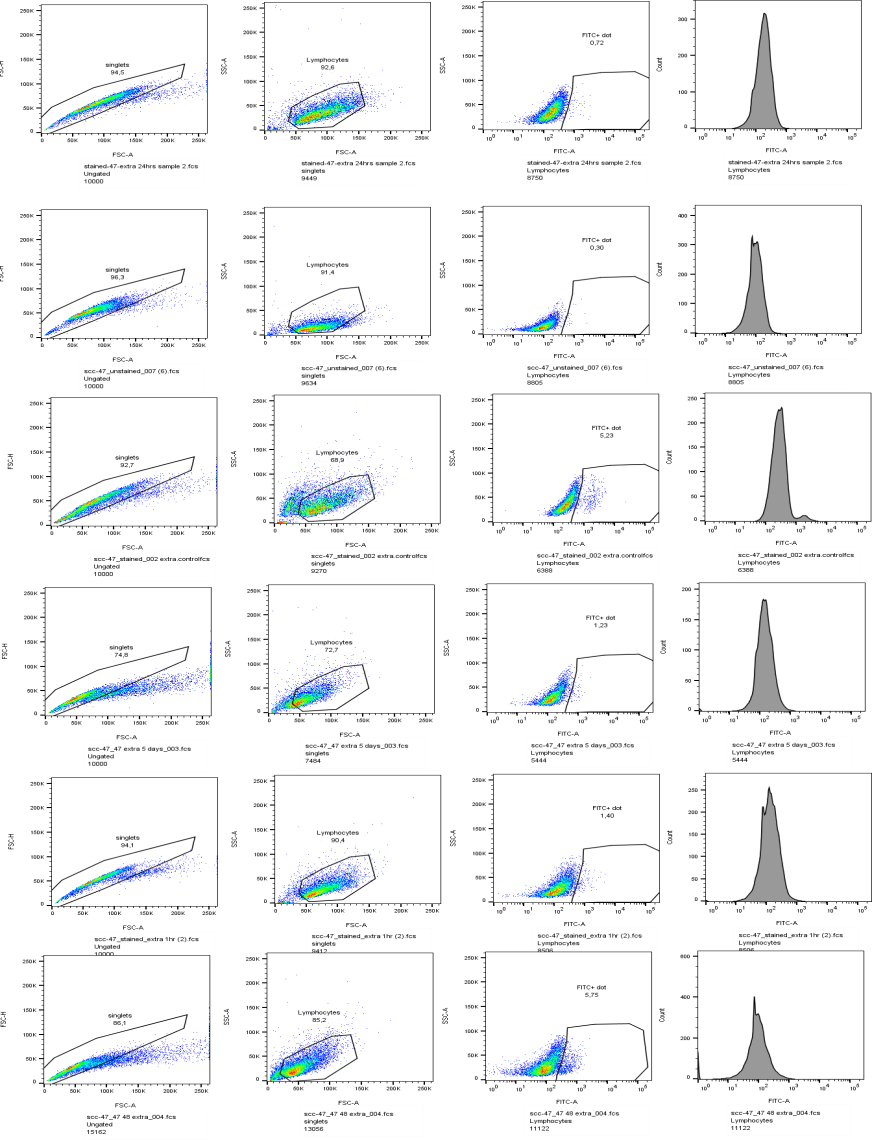
A B C D

# UM-SCC-47 extracellular


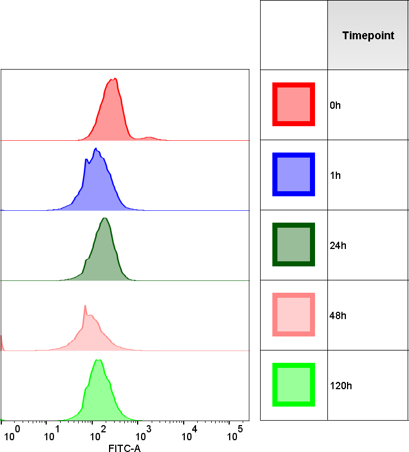


D


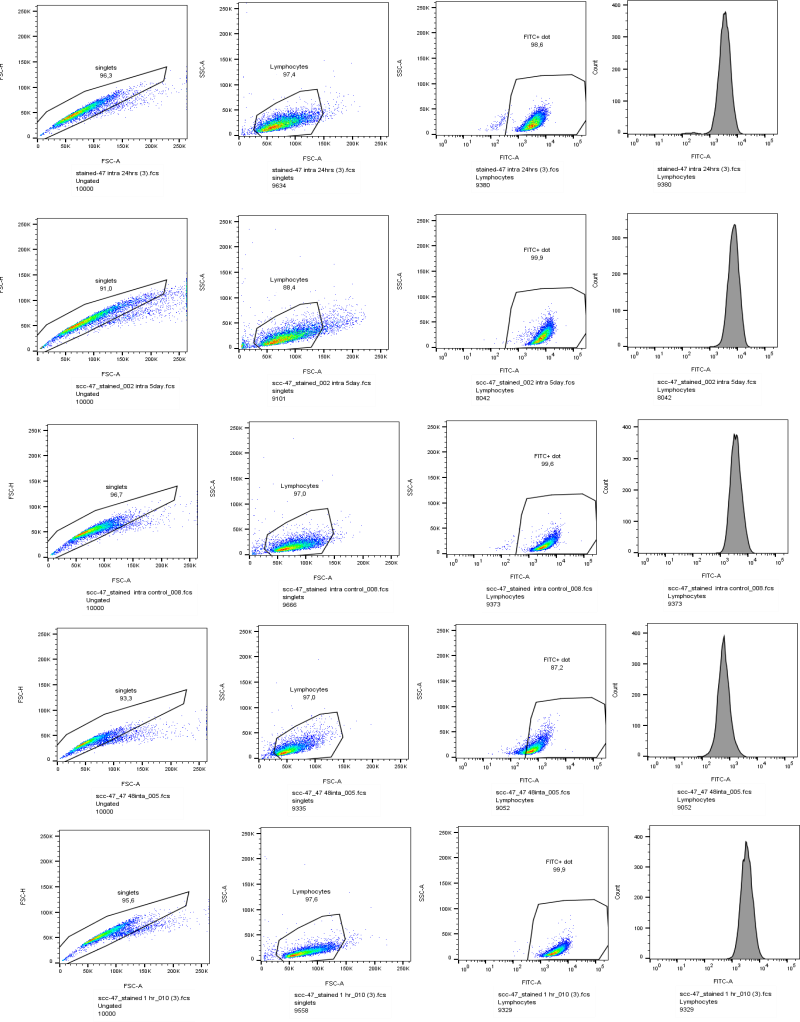
A B C D

# UM-SCC-47 intracellular


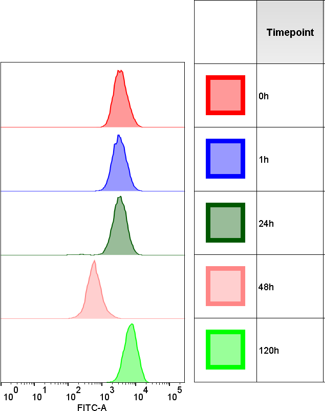


D


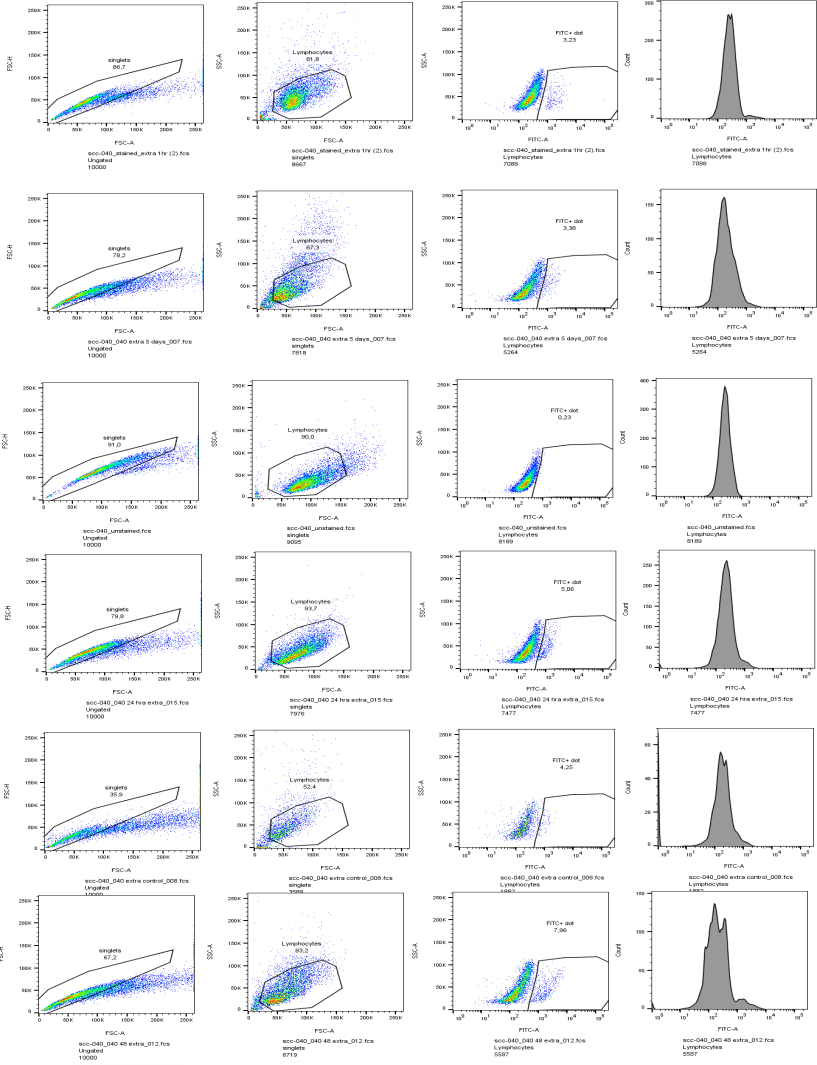
A B C D

# VU-SCC-040 extracellular


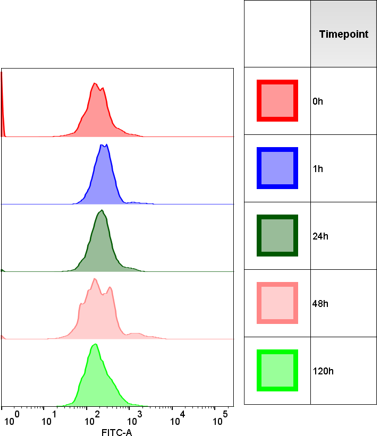


D


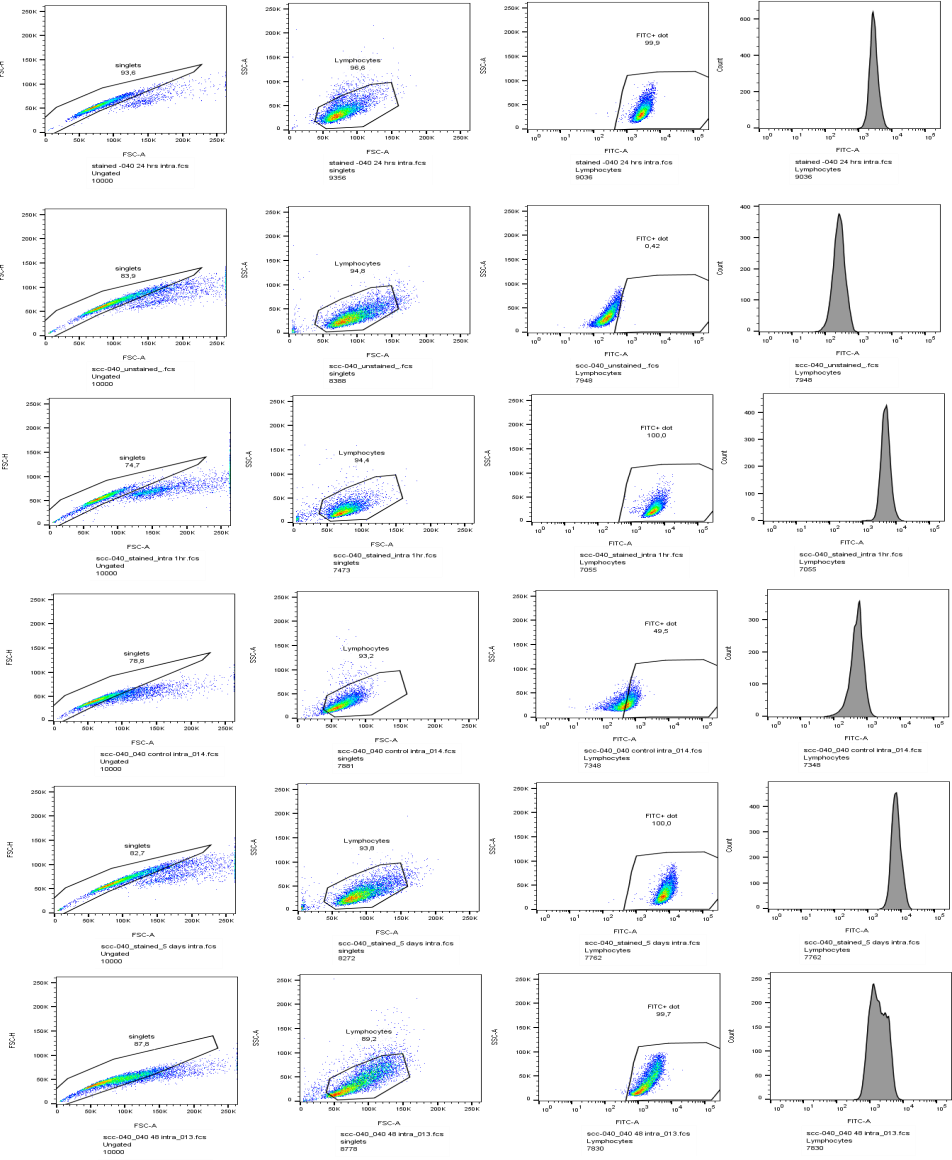
A B C D

# VU-SCC-040 intracellular


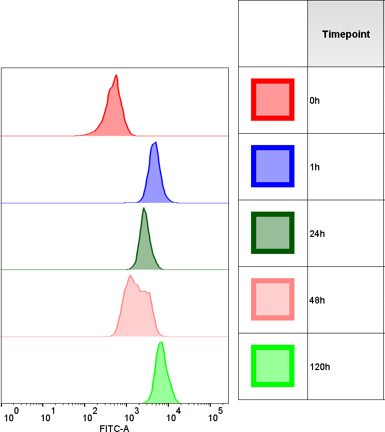


D
